# Frequency of Thrombolysis in Myocardial Infarction III Flow in Patients With Primary Percutaneous Coronary Intervention: Not All Culprit Vessels Are Completely Occluded in ST Elevation Myocardial Infarction

**DOI:** 10.7759/cureus.12036

**Published:** 2020-12-12

**Authors:** Muhammad Hussain, Rajesh Kumar, Ali Ammar, Syed Alishan, Atif S Muhammad, Fawad Farooq, Tahir Saghir, Naveedullah Khan, Syed N Hassan Rizvi, Tariq Ashraf

**Affiliations:** 1 Interventional Cardiology, National Institute of Cardiovascular Diseases, Karachi, PAK; 2 Adult Cardiology, National Institute of Cardiovascular Diseases, Karachi, PAK; 3 Cardiology, National Institute of Cardiovascular Diseases, Karachi, PAK; 4 Cardiology, Ziauddin Mefical University, Karachi, PAK

**Keywords:** coronary artery disease, st elevation myocardial infarction, primary percutaneous coronary intervention, infarct related artery, spontaneous revascularization, thrombolysis in myocardial infarction, vessel patency

## Abstract

Background

ST elevation myocardial infarction (STEMI) is classically characterized by total occlusion of the culprit coronary artery. However during primary percutaneous coronary intervention (PCI) thrombolysis in myocardial infarction (TIMI) 0 flow is not observed in all patients' culprit arteries in angiographic views. This study was conducted to find out the frequency of TIMI flow in acute STEMI patients in view of the above concept. The aim of this study was to evaluate the frequency of pre-procedural TIMI III flow in those patients who underwent primary PCI for acute STEMI in a public sector hospital in Karachi, Pakistan.

Methodology

This study is an audit of already saved data in the catheterization laboratory of the National Institute of Cardiovascular Diseases (NICVD), Karachi, that was collected prospectively from January 2016 to December 2018. These data were collected after taking consent from those patients who presented to hospital within 12 hours of symptoms and underwent primary PCI. Data were entered and analyzed on Statistical Package for the Social Sciences (SPSS) version 19 (IBM Corp., Armonk, NY, USA).

Results

A total of 8018 patients were included in this study who presented with STEMI and underwent primary PCI. Out of them 80.9% were males. Hypertension was the leading risk factor in 54.1% (4340) of patients. TIMI III flow was present in 11.4% of patients before primary PCI, while TIMI 0, I and II flow were present in 57.1%, 15.1%, and 16.3% of patients respectively (p<0.001). Fourteen percent of patients with TIMI III flow were of age group 51 to 60 years. Among those who had TIMI III flow, 11.2% were those with door to balloon time of <90 minutes. In 11% of cases, left anterior descending (LAD) artery had TIMI III flow as compared to other vessels (p<0.001). The length of the lesion was significantly smaller in patients who had TIMI III flow compared to those who had TIMI 0-II flow.

Conclusions

This study revealed that not all patients with acute STEMI had totally occluded culprit coronary artery but some of them had angiographic TIMI I-III flow in the infarct-related artery. Further studies are needed to find the reason for re-establishment of flow in the culprit vessel in STEMI patients before PCI.

## Introduction

Ischemic heart disease (IHD) is one of the major global causes of death. It is predicted that in 2020 cardiovascular disease is the leading cause of morbidity and mortality [1]. There are different treatment modalities for the treatment of IHD and percutaneous coronary intervention (PCI) is one of the best choices. In an acute setting, i.e. ST elevation myocardial infarction (STEMI), primary percutaneous coronary intervention (pPCI) has a better outcome and is preferred over other treatment options [2]. In the DANish Acute Myocardial Infarction 2 (DANAMI-2) trial, primary PCI was proven superior to thrombolytic therapy [3]. In primary PCI most important factor is blood flow in coronary arteries, which is measured by angiographic grading tool i.e. thrombolysis in myocardial infarction (TIMI) flow grade.

It is observed that patients with STEMI have different TIMI flow grades in coronary arteries; most of these patients have a completely occluded coronary artery but some patients have TIMI III flow in the coronary artery [4] which is sometimes due to spontaneous revascularization (SR) or due to medications (aspirin, GP IIb IIIa inhibiter, anticoagulation) given before pPCI. TIMI III flow is related to better outcomes of pPCI and less major adverse cardiovascular events (MACE) are observed in these patients [5]. It has been also studied that TIMI III flow or ST resolution (SR) has greater evidence of myocardial salvage with less chance of left ventricular failure and survival benefits i.e. 39% reduction in mortality [6,7].

In the ST-segment elevation acute myocardial infarction (ASSENT-4) trial, it was analyzed that those patients who have pre-PCI TIMI III flow have also higher TIMI flow after PCI, improved SR and better clinical outcome after 90 days of pPCI [8]. Thus TIMI III flow can bring extra benefits for the patients with STEMI and less chance of slow flow in coronary arteries after pPCI.

Frequency of TIMI III flow in pre-pPCI ranges between 10-30% [9,10]. This study was conducted to find out the frequency of TIMI III flow in acute STEMI. Recognition of those predictors which bring spontaneous revascularization in the culprit artery will provide new treatment information for STEMI and bring prognostic information.

## Materials and methods

This study was a retrospective observational study that was done after approval from the ethical review committee (ERC #36/2020). It was done by reviewing hospital registry data that was collected from January 1, 2016 December 31, 2018 by the interventional cardiology department of the National Institute of Cardiovascular Diseases (NICVD), Karachi, Pakistan. These data were collected by the cardiologist after taking consent from those patients who presented to hospital within 12 hours with symptoms of STEMI and underwent pPCI and questionnaire proforma was filled. All data were extracted from these pre-filled questionnaire proforma that include patient’s demographics and other variables like age, gender, risk factors, symptoms onset to hospital arrival and door to balloon time, number of vessels involved, culprit vessel, and lesion length, etc. Frequency of TIMI flow was calculated as TIMI 0, I, II and III, which are classified as follows: TIMI 0 flow, no perfusion to the myocardium after the occlusion of coronary artery (penetration without perfusion); TIMI 1 flow, incomplete filling of the distal coronary bed due to faint antegrade flow (partial perfusion); TIMI 2 flow, delayed and sluggish blood flow but with complete filling of the distal coronary bed (partial reperfusion); and TIMI 3 flow, normal blood flow in the coronary artery that fills the distal bed completely.

Two main groups were formed according to TIMI flow; one with TIMI 0-II flow and the other with TIMI III flow. Extracted data were entered into Statistical Package for the Social Sciences (SPSS) version 19 (IBM Corp., Armonk, NY, USA) and appropriate mean ± standard deviation (SD) or frequency percentages as descriptive statistics were computed for the quantitative and qualitative variables respectively. Bivariate analysis with Chi-square test and t-test/Mann-Whitney U test were performed to assess the associated factors with patency of coronary arteries. P-value ≤ 0.05 was considered statistical significant.

## Results

In this study, 8018 patients were included who presented with STEMI and underwent pPCI within 12 hours of onset of symptoms. In Table 1, baseline demographic, clinical, and angiographic characteristics have been reported. Most of the patients in this study were male (80.9%). The majority of patients with STEMI were in the age group of 51-60 years. Hypertension was the leading risk factor in 54.1% of patients followed by diabetes mellitus (DM) in 30.7% and smoking in 27.9% of the patients. The majority (67.2%) of patients were those who presented to the hospital after 120 minutes of onset of symptoms.

**Table 1:** Baseline demographic, clinical, and angiographic characteristics SD = standard deviation, TIMI = thrombolysis in myocardial infarction

Characteristics	Base
Total	8018
Gender	
Male	80.9% (6489)
Female	19.1% (1529)
Age	
Mean ± SD years	54.85 ± 11.42
Up to 40 years	11.6% (929)
41 to 50 years	28.4% (2275)
51 to 60 years	34% (2726)
More than 60 years	26% (2088)
Symptom onset to hospital arrival time	
Mean ± SD minutes	218.01 ± 133.91
≤ 120 minutes	32.8% (2630)
> 120 minutes	67.2% (5388)
First Medical Contact (FMC) to Device time	
Mean ± SD minutes	75.91 ± 55.26
≤ 90 minutes	75.9% (6089)
> 90 minutes	24.1% (1929)
Total ischemia Time	293.92 ± 143.99
Pre Procedural TIMI Flow Rate	
0	57.1% (4579)
I	15.1% (1212)
II	16.3% (1309)
III	11.4% (918)
Number of Vessels Involved	
Single vessel (SVD)	38.3% (3067)
Two vessels (2VD)	31.8% (2551)
Three vessels (3VD)	29.6% (2374)
None	0.3% (26)
Infarct Related Artery	
Left anterior descending (LAD)	54.7% (4383)
Right coronary artery (RCA)	32.9% (2635)
Left circumflex (LCX)	11.1% (886)
Ramus	0.2% (17)
Left main (LM)	0.2% (16)
Diagonal	0.6% (52)
None	0.3% (22)
Other	0.1% (7)

Pre-procedural TIMI flow in 57.1% of patients was TIMI 0 while in 11.4% of patients it was TIMI III flow. Thirty-eight percent (38%) of our patients had single-vessel coronary artery disease while 29.6% had triple-vessel disease. Left anterior descending (LAD) was the culprit artery in 54.7% of cases as compared to other coronary arteries.

In Table 2 TIMI flow rate was calculated by demographic, clinical, and angiographic characteristics. Patients were divided into two groups on the basis of TIMI flow (TIMI 0-II and TIMI III). Younger patients up to 40 years of age had high TIMI III flow rate as compared the older age group (14.1% vs. 11.8% respectively; p=0.024). Symptoms onset to hospital arrival time remarkably affected the TIMI flow. Those patients who came to the hospital within 120 minutes of symptoms onset had a higher rate of TIMI III flow as compared to those who came after 120 minutes (12.6% and 10.9% respectively; p=0.026). Patients who had <90 minutes of first medical contact (FMC) to device time had a higher rate of TIMI 0-II as compared to those who had FMC to device time >90 minutes (11.2% and 12.4%, respectively). Among major coronary arteries, left circumflex (LCX) had a higher rate of TIMI III (12.2%) flow as compare to LAD and right coronary artery (RCA). However among all epicardial vessels, diagonal artery had maximum pre-procedure patency rate (21.2%; p<0.001) as compared to all other coronary arteries.

**Table 2:** Pre-procedure TIMI III flow rate by demographic, clinical, and angiographic characteristics SD = standard deviation, TIMI = thrombolysis in myocardial infarction

Characteristics	Base (N)	TIMI III Flow Rate (%)		
		TIMI 0-2	TIMI 3	P-value
Total	8018	88.6% (7100)	11.4% (918)	-
Gender				
Male	6489	88.6% (5750)	11.4% (739)	0.725
Female	1529	88.3% (1350)	11.7% (179)	
Age				
Mean ± SD years	54.8 ± 11.4	54.9 ± 11.3	54.2 ± 12.3	0.06
Up to 40 years	929	85.9% (798)	14.1% (131)	0.024*
41 to 50 years	2275	88.2% (2007)	11.8% (268)	
51 to 60 years	2726	89.5% (2441)	10.5% (285)	
More than 60 years	2088	88.8% (1854)	11.2% (234)	
Risk Factors				
Diabetes Mellitus	2464	88.2% (2174)	11.8% (290)	0.549
Hypertension	4340	88.9% (3858)	11.1% (482)	0.294
Smokers	2237	88% (1969)	12% (268)	0.353
Symptom Onset to Hospital Arrival time				
Mean ± SD minutes	218 ± 133.9	219.2 ± 134.3	208.6 ± 130.4	0.015*
≤ 120 minutes	2630	87.4% (2299)	12.6% (331)	0.026*
> 120 minutes	5388	89.1% (4801)	10.9% (587)	
First Medical Contact (FMC) to Device time				
Mean ± SD minutes	75.9 ± 55.3	75.4 ± 54.1	80 ± 63.1	0.211
≤ 90 minutes	6089	88.8% (5410)	11.2% (679)	0.137
> 90 minutes	1929	87.6% (1690)	12.4% (239)	
Total Ischemia Time	293.9 ± 144	294.6 ± 144.3	288.6 ± 141.4	0.242
Number of Vessels Involved				
Single vessel (SVD)	3067	88.2% (2706)	11.8% (361)	
Two vessels (2VD)	2551	89.6% (2286)	10.4% (265)	
Three vessels (3VD)	2374	88.5% (2101)	11.5% (273)	
None	26	26.9% (7)	73.1% (19)	
Infarct Related Artery				
Left anterior descending (LAD)	4383	89% (3902)	11% (481)	<0.001*
Right coronary artery (RCA)	2635	88.6% (2335)	11.4% (300)	
Left circumflex (LCX)	886	87.8% (778)	12.2% (108)	
Ramus	17	94.1% (16)	5.9% (1)	
Left main (LM)	16	87.5% (14)	12.5% (2)	
Diagonal	52	78.8% (41)	21.2% (11)	

## Discussion

The remarkable result of this study showed that not all culprit vessels in STEMI are completely occluded. It was calculated by measurement of TIMI flow rate in the culprit vessel. It was found that 11.4% of STEMI patients have TIMI III flow before pPCI, which is less than the previous studies (14-22%) [11-13]. It is well documented in the literature that myocardial infarction occurs due to plaque rupture that leads to thrombus formation in the coronary artery and that is the ultimate pathophysiology of the STEMI. Thrombus formation occurs due to imbalance between the coagulation pathway and fibrinolytic system [14] and ultimately endogenous fibrinolytic system dominate and spontaneous revascularization of the culprit vessel takes place [15,16]. There are multiple endogenous factors responsible for spontaneous revascularization. These factors potentiate the process of auto thrombolysis and include hepsin, cathepsin and tissue plasminogen activators u-PA, t-PA, release from white blood cells and endothelial cells [17].

Another important finding of this study was that those patients who had less FMC to device time have less TIMI III flow rate as compared to those who have >90 minutes. The possible explanations for this are the endogenous and exogenous mediators. The exogenous mediators are medications given before pPCI [18]. These medications are antithrombotic, e.g. aspirin, clopidogrel, ticagrelor, and prasugrel, and anticoagulation e.g. heparin and enoxaparin [19]. The values of clopidogrel and tirofiban to maintain TIMI III flow before pPCI were studied in STEMI patients and it was found that they increase the chance of spontaneous revascularization in such patients [20]. Aspirin and clopidogrel require almost two to four hours to achieve maximum effects,however, the time between the loading dosage of these medicines and pPCI was less than one hour so these drugs could not achieve peak therapeutic effects. There are some other medications that have rapid onset of action e.g. ticagrelor and prasugrel [21,22].

The relation between TIMI III flow was also related to some other factors, e.g. age and gender; young women have less coronary artery disease and they have more often TIMI III flow before PCI compared to similarly aged men [23]. In this study gender had no significant difference in TIMI flow but the younger age group had more pre-procedure TIMI flow rate as compared to the older age group.

It was also observed in this study that smoking has no significant effect on the angiographic TIMI flow rate in the culprit artery, however some studies revealed that pre-procedure TIMI flow rate was better in current smokers [24]. This study also showed that diabetes mellitus has no effect on pre-procedural TIMI flow rate. In other studies, diabetes mellitus has directly affected pre-pPCI TIMI flow rate but post-procedure there is no difference in TIMI flow rate in diabetic and non-diabetic patients [25]. It was found in this study that symptom onset to hospital arrival time has an effect on the pre-pPCI TIMI flow rate; those patients who came to the hospital earlier have a higher rate of TIMI III flow rate as compared to later comers. The relationship between the late arrival to hospital and TIMI III flow was proven by other studies; it was found that if total ischemic time is higher then there is less chance of TIMI III flow rate in STEMI patients [26,27].

It was found that the LAD is the most commonly involved artery in STEMI and this result was comparable to other studies [28,29]. Among all major coronary arteries, LCX had more TIMI III flow rate and the same result was found in another study [29]. This study also showed that TIMI III flow was more in small-length culprit lesions, however there is not enough data that support this finding.

This is a retrospective, non-randomized design study; we only audited the frequency of pre-procedure TIMI III flow in patients coming to our hospital for pPCI. We could not assess the causes of pre-procedural TIMI III flow; pre- and post-procedure left ventricular function and its impact on patient outcome can be evaluated by further prospective studies.

## Conclusions

In this study we concluded that not all patients with STEMI have completely occluded culprit artery, which is proven by other studies. Further studies are required to find the relevant factors which are responsible for high TIMI III flow rate and to manage these factors so that spontaneous revascularization in STEMI can be achieved and ultimately can provide better outcome to the patients.
